# Adiposity Is Related to Inflammatory Disease Activity in Juvenile Idiopathic Arthritis

**DOI:** 10.3390/jcm10173949

**Published:** 2021-08-31

**Authors:** Gisela Diaz-Cordovés Rego, Esmeralda Núñez-Cuadros, Natalia Mena-Vázquez, Soledad Aguado Henche, Rocío Galindo-Zavala, Sara Manrique-Arija, Laura Martín-Pedraz, Rocio Redondo-Rodríguez, Francisco Javier Godoy-Navarrete, Antonio Fernández-Nebro

**Affiliations:** 1 Instituto de Investigación Biomédica de Málaga (IBIMA), 29010 Málaga, Spain; gisela.d.cordoves@hotmail.com (G.D.-C.R.); sarama_82@hotmail.com (S.M.-A.); rocioredondo91@hotmail.com (R.R.-R.); fjgodoynavarrete@gmail.com (F.J.G.-N.); afnebro@gmail.com (A.F.-N.); 2 UGC de Reumatología, Hospital Regional Universitario de Málaga, 29009 Málaga, Spain; 3 UGC de Pediatría, Hospital Regional Universitario de Málaga, 29009 Málaga, Spain; esmenunez@gmail.com (E.N.-C.); rociogalin@hotmail.com (R.G.-Z.); Pedraz88@hotmail.com (L.M.-P.); 4 Departamento de Anatomía y Embriología Humana, Facultad de Medicina, Universidad de Alcalá de Henares, 29009 Madrid, Spain; soledad.aguado@uah.es; 5 Departamento de Medicina, Universidad de Málaga, 29010 Málaga, Spain

**Keywords:** juvenile idiopathic arthritis, adiposity, inflammatory disease activity

## Abstract

Objective: To identify factors associated with the higher proportion of fatty tissue and overweight/obesity observed in patients with juvenile idiopathic arthritis (JIA). Patients and methods: We performed a cross-sectional study of 80 JIA patients aged 4–15 years with 80 age- and sex-matched healthy controls. Body composition was assessed using dual-energy x-ray absorptiometry. The 27-joint Juvenile Arthritis Disease Activity score (JADAS27) was calculated. Two multivariate models were constructed to identify factors associated with overweight/obesity and fat mass index (FMI). Results: No differences were found between cases and controls in body mass index (BMI) or body composition. However, compared with controls, patients with a high inflammatory activity (JADAS27 > 4.2 for oligoarticular JIA or >8.5 for polyarticular disease) had higher values for BMI (*p* = 0.006); total fat mass (*p* = 0.003); FMI (*p* = 0.001); and fat in the legs (*p* = 0.001), trunk (*p* = 0.001), and arms (*p* = 0.002). The factors associated with overweight/obesity in patients were the duration of therapy with biological drugs, measured in months (OR [95% CI] = 1.12 [1.02–1.04]; *p* = 0.037), and physical activity (OR [95% CI] = 0.214 [0.07–0.68]; *p* = 0.010), while the factors associated with FMI were age (β [95% CI] = 0.30 [0.17–1.41]; *p* = 0.014), JADAS27 (β [95% CI] = 0.45 [0.16–1.08]; *p* = 0.009), and physical activity (β [95% CI] = −0.22 [−5.76 to 0.29]; *p* = 0.031). Conclusion: Our study revealed no differences between JIA patients with well-controlled disease and low disability and the healthy population in BMI or body composition. Furthermore, the association observed between inflammatory activity and adiposity could be responsible for poorer clinical course.

## 1. Introduction

Juvenile idiopathic arthritis (JIA) is the most frequent chronic inflammatory rheumatic disease in childhood. It encompasses a heterogeneous group of inflammatory conditions comprising various categories or subtypes whose common clinical manifestation is arthritis [1,2]. New criteria intended for classification are currently being validated. These could identify more homogeneous groups and better distinguish between childhood rheumatic diseases and those that represent the earlier onset of adult disease [3]. Their etiology and pathogenesis are complex and, as in rheumatoid arthritis (RA), they have an immunologic basis in which proinflammatory cytokines are overexpressed [4,5]. The objectives of treatment include disease control, appropriate growth and development, and reduced long-term comorbidity [6].

Delayed growth [7,8,9], reduced muscle and bone mass [10,11,12,13], and nutritional deficiency [14,15,16] are the most commonly reported long-term complications in the polyarticular [17], systemic [6,18,19], and oligoarticular [20] subtypes. Some of these abnormalities have been associated with more marked disease activity, reduced physical exercise [10], and more frequent use of glucocorticoids [8,21]. In contrast, the inclusion of biologics in therapy has improved disease control, growth, and body composition [2,6,7,19,21,22,23]. Nevertheless, recent years have seen reports of overweight and obese children affected by the disease [17,24,25,26,27]. The main potential causes of this phenomenon include greater calorie intake [28], reduced physical exercise, disability [19,29], inflammation of lower limb joints [4], disease subtype, treatments used [26], or a combination of all these factors [17].

Today, it is known that fatty tissue behaves as a relevant inflammatory tissue in the pathogenesis and persistence of inflammatory activity. In hyperadiposity, activated adipocytes release increased quantities of proinflammatory mediators and adipokines, including TNF-α, IL-12, and IL-6 [30]. In fact, some studies of RA and psoriatic arthritis in adults have shown that fatty tissue plays a role in the disease course and response to therapy [31,32]. In patients with RA, the accumulation of fatty tissue is accompanied by reduced muscle mass, owing to the effect of proinflammatory cytokines and muscular atrophy resulting from disuse. This change may go undetected if we only take into account body mass index (BMI), which does not discriminate between fat and muscle components [30,33,34,35,36]. Nevertheless, BMI is the most widely cited evaluation method and the one most discussed in the medical literature for classifying patients as overweight or obese [37]. Dual-energy x-ray absorptiometry (DXA) is the gold standard tool for assessing body composition [34,38]. However, owing to difficulties with the accessibility of this technology, few studies have identified the factors associated with greater fatty mass in body composition using DXA in patients with JIA and the possible association between fatty tissue, BMI, and inflammatory activity [4,18]. Consequently, the main objective of our study was to identify those factors associated with a higher proportion of fatty tissue and overweight/obesity in patients with JIA assessed by DXA.

## 2. Materials and Methods

### 2.1. Design

We performed an observational, cross-sectional study of a series of patients with controlled JIA and an external control group of healthy subjects matched for age and sex. The study was approved by the Clinical Investigation Ethics Committee of Hospital Regional Universitario de Málaga, Malaga, Spain (HRUM) (4/14-PIA 2). Written informed consent was provided by the participants or their parents before participation. 

### 2.2. Study Population

A total of 80 patients were recruited between 2014 and 2015. Patients were aged between ≥4 and 15 years and had JIA according to the classification criteria of the International League of Associations for Rheumatology [1], and were followed at the Pediatric Rheumatology Department of HRUM. 

In order to ensure as homogenous a group as possible, we excluded patients with exclusively monoarticular disease (very low inflammatory activity), JIA associated with enthesitis (low number), and psoriatic and nondifferentiated arthritis (heterogeneous). We also excluded cases of JIA with active infection or other significant comorbidity of the nervous or endocrine system (hypo- or hyperthyroidism, diabetes mellitus), liver, kidney, or gastrointestinal tract that could also be the cause of systemic disease or interfere with the interpretation of the findings for the cases. Since uveitis was considered another symptom of JIA, it was not considered an exclusion criterion. 

The controls were selected between 1998 and 2003 from a population sample of Spanish individuals in Alcalá de Henares comprising 1113 healthy Caucasian volunteers (397 men and 716 women) aged <80 years [39]. The exclusion criteria for healthy controls were similar to those of patients. Both samples, cases and controls, were divided into 16 age groups at intervals of 5 years to facilitate matching. There were 193 controls (101 boys and 92 girls) aged between 4 and 16 years; this enabled consecutive matching with the cases by age and sex in a ratio of 1:1. 

### 2.3. Study Protocol

#### 2.3.1. Cross-Sectional Evaluation

After signing the informed consent document, all patients were interviewed and examined and then asked to complete a predesigned questionnaire for purposes of data collection. Disease activity indices were calculated, biological samples were taken after a 12- to 16-h fast before 10:00 a.m., and a whole-body composition study was performed using DXA. All data for cases and controls were entered into a database for subsequent analysis.

#### 2.3.2. Retrospective Evaluation

Patients with JIA were usually followed according to a pre-established protocol in the Pediatric Rheumatology Department of HRUM in line with their clinical status. Check-ups were generally every 3 or 6 months. At each check-up, physicians recorded clinical changes and potential adverse drug reactions, measured inflammatory activity, and readjusted treatment. 

### 2.4. Study Variables

The main outcome measures included anthropometric data and body composition recorded using whole-body DXA. Anthropometric measures included BMI (weight (kg) + height (m²)) and obesity, according to the World Health Organization’s (WHO) definition [40], which specifies the following categories: extremely underweight (BMI < −3 SD), underweight (BMI < −2 SD), normal weight (BMI < −2 and BMI +1 SD), overweight (BMI > +1 SD), and obese (BMI > +2 SD). Patients’ body compositions were measured using DXA (GE Lunar Prodigy) with CORE^TM^ 2006 software; controls were measured using a Norland XR-26 DXA system (version 2.3). The parameters measured included total mass (kg), fat mass (kg), lean mass (kg), fat and lean android and gynoid mass (kg), and appendicular fat and lean mass. The fat mass index (FMI) was defined as (fat mass (kg)/height squared (m^2^)), and the fat-free mass index (FFMI) as (fat mass (kg)/height squared (m^2^)) [41]. The skeletal muscle mass index was calculated based on the appendicular skeletal muscle mass in kilograms divided by the height in m^2^ [42,43].

### 2.5. Other Variables Recorded in Patients with JIA 

Data recorded for patients with JIA also included demographic data, clinical data, laboratory results, and treatment. The reference date was that of the cross-sectional study. Demographic variables included age in years, sex, and race. The characteristics of patients with JIA included the date of disease onset, duration (calculated from the diagnosis to the reference date), active uveitis at the reference date and during follow-up, and the following laboratory data at the reference date: rheumatoid factor (RF), which was considered positive if >20 IU/mL; antinuclear antibody (ANA), which was considered positive if the titer was >1/80; erythrocyte sedimentation rate (ESR), C-reactive protein (CRP), and high-sensitivity CRP (hsCRP). Activity was also assessed quantitatively using the 27-joint Juvenile Arthritis Disease Activity score (JADAS27) and classified as follows: (1) high inflammatory activity (JADAS27 > 4.2 for oligoarticular JIA and >8.5 for polyarticular JIA); (2) moderate activity (JADAS27 of 4.2–2.1 for oligoarticular JIA and 8.5–3.9 for polyarticular JIA); (3) mild activity (JADAS27 2–1.1 for oligoarticular JIA and 3.8–1.1 for polyarticular JIA); and (4) inactive (JADAS27 ≤ 1 for any subtype) [44,45,46]. For purposes of the analysis, categories 2, 3, and 4 were reclassified into a single group (not high activity). Disability was evaluated using the Children’s Health Assessment Questionnaire (CHAQ) [47]. Physical activity in children with JIA was measured using the International Physical Activity Questionnaire (IPAQ) adapted to the different age groups: PAQ-C (Physical Activity Questionnaire for Children) for children aged <12 years and PAQ-A (Physical Activity Questionnaire for Adolescents) for adolescents aged 12–17 years [48]. Similarly, current and previous conventional synthetic disease-modifying antirheumatic drugs (csDMARDs) and biologic DMARDs (bDMARDs) were recorded. Treatment with glucocorticoids was recorded, as were the mean and cumulative doses since onset of the disease. 

### 2.6. Statistical Analysis

A descriptive analysis was performed based on absolute frequencies and percentages for qualitative variables and mean ± standard deviation (SD) or median (interquartile range [IQR]). The normality of the distribution was confirmed using the Kolmogorov–Smirnov test. Patients and controls were compared using the Pearson χ^2^ test or the *t* test, as applicable. The correlation (Pearson R) between fat mass, FMI, and lean mass and the clinical characteristics of JIA was assessed. The χ^2^ and ANOVA or Kruskal–Wallis test (depending on normality) were applied to compare the main characteristics between 3 groups of patients: (I) patients with JIA and high inflammatory activity; (II) patients with JIA and mild-moderate inflammatory activity or inactive disease (assessed together); and (III) controls. Finally, 2 multivariate models were run, one based on logistic regression to identify factors associated with overweight/obesity according to BMI in patients with JIA, and another based on multiple linear regression to identify factors that were independently associated with FMI in patients with JIA. The variables entered into the models were those that had proven significant in the bivariate analysis and were of clinical interest. Statistical significance was set at *p* < 0.05. Given an alpha risk of 0.05 and a beta risk of 0.2 in a bilateral contrast, the sample size calculation showed that 78 patients and 78 controls were necessary to detect an expected significant difference in obesity of 31% for patients with JIA and 12.5% for controls [25]. Data entry and the statistical analysis were performed using R-commander (https://www.rcommander.com (accessed on 20 January 2021). 

## 3. Results

### 3.1. Epidemiological and Clinical-Laboratory Characteristics and Treatment

The study population comprised 80 patients with JIA and 80 healthy controls. Table 1 shows that both groups are well-matched for sex and age. Most cases were girls (70%), with a mean (SD) age of 10.2 (3.2) years and a mean (SD) duration of 6.5 years (3.7). Most of the patients were Caucasian (European ancestry, 77/80, 96.2%), followed by Amerindian Caucasians (2/80, 2.5%) and Black Africans (1/80, 1.2%). The most numerous clinical groups were persistent oligoarticular JIA (47%), followed by RF-negative polyarticular disease (24%). According to the JADAS27, inflammatory activity was well-controlled in most cases, and disease was inactive in 72% of the study population. Of the 15 patients with high activity, five had persistent oligoarticular JIA, five had extended oligoarticular disease, three had polyarticular disease, and two had systemic disease. The mean CHAQ (0.17) indicated low disability.

Almost all the patients (98.7%) received csDMARDs, especially methotrexate, and almost 50% needed bDMARDs. The cross-sectional study revealed that 25% of patients were in remission without treatment, 42/80 (52%), and were taking methotrexate, and only one third, 24/80 (30%), were taking bDMARDs, the most effective anti-tumor necrosis factor agents (anti-TNF).

Glucocorticoids had been taken at some point by 71 patients (89%), although only 7/80 (9%) were taking them on the reference date. The median (IQR) cumulative dose of glucocorticoids was 6.1 (2.8–14.6) mg/kg for a median (IQR) of 125.0 (66.0–179.0) days. Patients with systemic JIA took the highest cumulative doses of glucocorticoids (median (IQR), 40.8 (25.0–60.1) vs. 2.6 (1.0–6.1) mg/kg; *p* < 0.001) and for longer than the others (median (IQR), 342.0 (145.0–858.0) vs. 116.5 (53.7–154.5) days; *p* = 0.001).

### 3.2. Anthropometric Characteristics of JIA Patients Compared with Controls

Supplementary Table S1 shows the anthropometric characteristics and body composition by DXA for cases and controls. Both groups had a similar weight, height, and BMI, with most being a normal weight (84% vs. 87%; *p*= 0.515). Similarly, we found no differences in total fat mass (*p* = 0.449), total lean mass (*p* = 0.793), or in fat and lean mass in the legs, arms, and trunk. However, compared with patients with lower levels of inflammatory activity and healthy controls, patients with very active disease according to JADAS27 had higher values for BMI (*p* = 0.006), total fat mass (*p* = 0.003), FMI (*p* = 0.001), and fat mass in the legs (*p* = 0.001), trunk (*p* = 0.001), and arms (*p* = 0.002) (Table 2). The anthropometric characteristics and body composition of patients with JIA and high and low disease activity, excluding patients with systemic JIA showed similar results (Supplementary Table S2).

### 3.3. Factors Associated with Overweight/Obesity in Patients with JIA

Thirteen of 80 patients (16%) were overweight/obese (BMI > 1 SD) according to the WHO. Supplementary Table S3 shows the results of the bivariate analysis for patients with JIA who were and were not overweight/obese. Both groups had similar epidemiological and clinical characteristics and body compositions, as measured by DXA. The only difference was that, compared with normal weight patients, those with overweight/obesity had had inflammatory activity for longer (mean (SD), 116.7 (76.4) vs. 78.2 (37.1) months; *p* = 0.079), had higher hsCRP levels (mean (SD), 4.2 (2.9) vs 1.9 (3.3) mg/L; *p* = 0.048), and more frequently took bDMARDs (*n* (%), 7 (53.8) vs. 17 (25.4); *p* = 0.040) for longer (mean (SD), 37.1 (20.2) vs. 16.2 (14.4) months; *p* = 0.014). There were no differences in Anti-TNF-α, while overweight/obese patients had had Anti-IL6 taken from them more frequently; and patients who were not overweight/obese had had Anti-IL1 taken from them more frequently. However, no differences were found in the cumulative dose of glucocorticoids (mg/kg) between the groups (median (IQR), 11.2 (4.4–31.2) vs. 4.4 (2.4–14.4) mg/kg *p* = 0.102).

Table 3 shows the results of the multivariate logistic regression analysis for the dependent variable overweight–obese in patients with JIA. As can be seen, the presence of overweight/obesity was associated with a longer duration of therapy with bDMARDs and reduced physical activity. These factors would account for 24% of the variability in the presence of overweight/obese patients (R^2^ = 0.242). Another logistic regression model excluding patients with systemic JIA was constructed, which showed similar results (Supplementary Table S4).

### 3.4. Factors Associated with Fat and Lean Mass in Patients with JIA

Supplementary Table S5 shows the bivariate correlations between fat mass, FMI, and lean mass for patients with JIA, as well as clinical, laboratory, and therapy-related variables. A positive correlation was found between FMI and age, time since diagnosis, JADAS27, hsCRP, and the duration of therapy with bDMARDs. Similarly, a negative correlation was detected with physical activity. Correlations for fat mass were similar to those for FMI. As for lean mass, a positive correlation was observed for age, time since diagnosis, time spent taking csDMARDs, and physical activity. 

Table 4 shows the results of the multivariate linear regression analysis (dependent variable: FMI) in patients with JIA. FMI was independently associated with age, inflammatory activity (JADAS27), and physical activity. Therefore, FMI increased by a mean of 0.30 kg per year of the patient’s age and 0.44 kg per point increase in the JADAS27 score, and decreased 0.2 kg per point of physical activity. Another multivariate linear regression analysis excluding patients with systemic JIA was constructed, which showed similar results (Supplementary Table S6).

## 4. Discussion

In adult chronic arthritis, such as rheumatoid arthritis and psoriatic arthritis, obesity and adiposity have a negative effect on disease control and response to therapy [49]. However, few studies have evaluated the association between adiposity and/or obesity and inflammatory activity in JIA. Given that most of these studies base their evaluation on anthropometric values and not DXA values [17,24,26,27,50], they may not suitably reflect patients’ fat content. In fact, only one study addressed body composition analysis with DXA, although it was restricted to girls [27], and another used bioelectrical impedance analysis [17]. In our study, however, we used both anthropometric values and body composition by DXA, which is currently considered the gold standard for evaluating this parameter [51]. 

Consistent with findings from recent controlled studies [17,25] and noncontrolled studies [26,50], our results showed that patients with JIA did not differ from controls with respect to BMI. While the percentage of overweight and obese participants in our study was lower than reported elsewhere [24,25,27,28,31], differences in population [26,31], methodology, and the definitions of obesity used hampered comparison. The prevalence of obesity in childhood varies according to sex and age, being the highest in 9-year-old children (24.1%; 95% CI 23.9–24.3) [52]. In addition to methodological differences, 81% of the patients in our study had mild inflammatory activity or inactive disease; in fact, 25% of cases were in remission without treatment. This good clinical control could be explained by the low frequency we found for overweight and obese participants. The finding was consistent with the other results we reported, because patients with a high disease activity, according to JADAS27, had a higher BMI and higher total, trunk, and appendicular mass than patients with lower disease activity. In fact, the multivariate analysis revealed overweight/obesity to be associated with a longer duration of biologic therapy and reduced physical activity. These results agree with those reported by Schenck et al. [26], who observed a decrease in the prevalence of overweight–obese participants (with more obese patients) from 6.1% in 2003 to 1.7% in 2012; this occurred in parallel with better disease control and the availability of new treatments. Therefore, it seems logical to think that better control of disease activity would facilitate a return to normal physical activity in children and normal development. 

In contrast, Caetano et al. [27] found greater adiposity with DXA in girls with JIA that was not associated with inflammatory activity. However, the authors did not report the degree of control of inflammation in their sample and did not apply JADAS27, which was the most accurate parameter for inflammatory activity in JIA [53]. 

Furthermore, our study analyzed the doses of glucocorticoids used since the onset of the disease and found that the mean dose was low, although there was no association with fatty mass or overweight–obese values. This finding could be explained by the low number of patients with systemic JIA evaluated in the study (*n* = 80), compared with that of Schenck et al. (*n* = 5667) [26], whose data source was a registry of patients with juvenile rheumatic disease in Germany including more patients with systemic JIA. 

The association we found between the duration of therapy with bDMARDs, and overweight–obese participants could be explained by the greater need for treatment arising from more severe disease. Likewise, it has been described in other studies that for adult patients with JIA in clinical remission, present subclinical signs of inflammation showed in an increase in inflammatory cytokines and cardiovascular risk [54].

Finally, our study is subject to a series of limitations. First, the controls were not ideal, because their study was performed using a different DXA with a significant time gap between collection of controls and patient data. While it was not our main objective to analyze differences between the two groups, the comparison was based on one of the groups as a reference, since both belonged to similar populations. One of the strengths of our study was that we analyzed in detail the effect of the drugs, including glucocorticoids taken from onset. Ours was also the first study to show an association between body composition measured by DXA and an association according to the inflammatory activity in JIA based on JADAS27; although some variables that could have influenced in fatty tissue and overweight–obese participants were not collected, such as family participation levels, socioeconomic status and food insecurity. Furthermore, we included a sample of patients with JIA that was both representative and more homogeneous, since the subtypes psoriatic arthritis, arthritis–enthesitis, and monoarticular disease were excluded [3]. A comparison of patients without inflammatory activity versus a group of patients with different inflammatory activity was made, instead of a more granular analysis of inflammation, to be able to see the effect of any degree of inflammation on fat tissue. The cross-sectional design of the study did not establish a causal relationship between obesity and inflammation in patients with JIA, only association. Notwithstanding, a prospective cohort study with a longer observation period would have been much better and yielded some conclusions about causality. Therefore, a longitudinal approach with patients used as your own controls is an approach that we should take into account for future studies.

## 5. Conclusions

In conclusion, our study shows that patients with well-controlled JIA and a low disability may not differ from the healthy population in terms of body composition and anthropometric measurements. Furthermore, an association was observed between inflammatory disease and adiposity, which could be responsible for a poorer clinical course. New studies on this subject are necessary, as are strategies for intervention in the management of affected patients. 

## Figures and Tables

**Table 1:** Characteristics of patients with JIA and controls on the reference date and during follow-up.

Variable	JIA (*n* = 80)	Controls (*n* = 80)	*p* Value
Epidemiological			
Sex, female, *n* (%)	56 (70.0)	57 (71.3)	0.862
Age, mean (SD)	10.7 (3.2)	10.2 (3.2)	0.893
Race, Caucasian *n* (%)	77 (96.3)	80 (100)	0.080
Clinical laboratory			
Time since diagnosis of JIA, years, mean (SD)	6.5 (3.7)		
Type of JIA			
Systemic, *n* (%)	9 (11.3)		
Oligoarticular persistent, *n* (%)	38 (47.5)		
Oligoarticular extended *n* (%)	13 (16.3)		
Polyarticular RF-positive, *n* (%)	1 (1.3)		
Polyarticular RF-negative, *n* (%)	19 (23.8)		
RF +, *n* (%)	1 (1.2)		
ANA +, *n* (%)	15 (18.7)		
Uveitis during follow-up, *n* (%)	20 (25.0)		
Inflammatory activity			
CRP (mg/L), mean (SD)	4.8 (9.5)		
hsCRP (mg/L) median (IQR)	0.62 (0.0–16.8)		
ESR (mm/h), mean (SD)	8.8 (7.3)		
NPJ (0–27), mean (SD)	0.25 (0.6)		
NIJ (0–27), mean (SD)	0.21 (1.2)		
JADAS27, mean (SD)	2.0 (4.0)		
High activity, *n* (%)	15 (18.8)		
Mild-moderate, *n* (%)	7 (8.8)		
Inactive, *n* (%)	58 (72.5)		
CHAQ, mean (SD)	0.17 (0.4)		
Treatment			
Current treatment			
csDMARD, *n* (%)	42 (52.5)		
bDMARD, *n* (%)	24 (30.0)		
Anti-IL-1, *n* (%)	4 (16.7)		
Anti-IL-6, *n* (%)	2 (8.3)		
Anti TNF-α, *n* (%)	18 (75.0)		
Duration csDMARDs (months), mean (SD)	51 (37.5)		
Duration bDMARDs (months), mean (SD)	19.7 (28.4)		
Duration all DMARDs (months), mean (SD)	55.8 (38.0)		
Cumulative dose of glucocorticoids (mg/kg), median (IQR)	6.1 (2.8–14.6)		

Abbreviations: JIA, juvenile idiopathic arthritis; SD, standard deviation; RF, rheumatoid factor; ANA, antinuclear antibodies; CRP, C-reactive protein; hsCRP, high-sensitivity C-reactive protein; ESR, erythrocyte sedimentation rate; NPJ, number of painful joints; NIJ, number of inflamed joints; JADAS27: Juvenile Arthritis Disease Activity Score; CHAQ: Childhood Health Assessment Questionnaire; csDMARD, conventional synthetic disease-modifying antirheumatic drug; bDMARD, biologic disease-modifying antirheumatic drug. +, positive.

**Table 2:** Anthropometric characteristics and body composition of patients with JIA and high and low disease activity according to JADAS27 vs. controls on the reference date.

Anthropometric Characteristics	JIA High Activity (*n* = 15)	JIA Not High Activity * (*n* = 65)	Controls (*n* = 80)	*p* Value
Anthropometric characteristics				
Weight, mean kg (SD)	45.0 (12.8)	36.9 (13.7)	38.7 (14.3)	0.175
Height, mean cm (SD)	149.8 (13.9)	144.0 (19.0)	143.0 (19.7)	0.581
BMI	20.8 (6.0)	17.6 (3.3)	18.1 (2.9)	0.006
Classification (WHO)				0.137
Normal weight, *n* (%)	11 (73.3)	56 (86.2)	71 (88.8)	
Overweight/Obesity, *n* (%)	4 (26.7)	9 (13.8)	9 (11.3)	
Body composition by DXA				
SAMMI, mean (SD)	5.4 (1.1)	5.0 (0.9)	4.3 (0.7)	<0.001
Total fat mass (kg), mean (SD)	18.1 (13.2)	9.7 (6.9)	12.2 (7.5)	0.003
FMI (kg/m^2^), mean (SD)	8.7 (4.6)	4.7 (2.8)	5.6 (2.6)	0.001
Total lean mass (kg), mean (SD)	28.5 (11.3)	24.8 (8.7)	25.0 (8.8)	0.396
FFMI (kg/m^2^), mean (SD)	13.0 (2.1)	12.0 (2.1)	11.9 (1.5)	0.160
Fat mass arms (kg), mean (SD)	1.6 (1.4)	0.8 (0.8)	1.1 (0.6)	0.002
Fat mass legs (kg), mean (SD)	6.7 (5.4)	3.7 (2.6)	3.7 (2.3)	0.001
Fat mass trunk (kg), mean (SD)	8.3 (8.1)	3.9 (3.4)	5.2(3.0)	0.001
Lean mass arms (kg), mean (SD)	2.5 (1.3)	2.3 (1.2)	2.1 (0.7)	0.117
Lean mass legs (kg), mean (SD)	8.9 (4.1)	7.9 (3.0)	7.3 (3.3)	0.192
Lean mass trunk (kg), mean (SD)	13.6 (5.4)	11.1 (4.1)	10.5 (4.0)	0.078
Physical activity				
PAQ-C/PAQ-A (score), mean (SD)	2.6 (0.7)	2.7 (0.6)		0.805

Abbreviations: JIA, juvenile idiopathic arthritis; SD, standard deviation; kg, kilogram; BMI, body mass index; DXA, Dual-energy x-ray absorptiometry; FMI, fat mass index; FFMI, fat-free mass index; SAMMI, skeletal appendicular muscle mass index; PAQ-C/PAQ-A, Physical Activity Questionnaire (children and adolescents). * No high activity includes patients with moderate and mild activity and inactive patients according to JADAS27; ±χ^2^, ANOVA, or Kruskal–Wallis.

**Table 3:** Logistic regression model of factors associated with overweight/obesity in patients with JIA.

Variable	Univariate OR (95% CI)	Multivariate OR (95% CI)	*p* Value
Age, years	0.898 (0.750, 1.075)		
Sex, female	0.429 (0.127, 1.447)		
Disease duration, months	1.030 (0.878, 1.209)		
JADAS27	1.006 (0.999, 1.013)		
Duration of bDMARD, months	1.022 (1.003, 1.041)	1.123 (1.020, 1.042)	0.037
hsCRP, mg/L	1.123 (1.009, 1.268)		
Physical activity, PAQ-C/PAQ-A, mean (SD)	0.422 (0.157, 1.128)	0.214 (0.067, 0.681)	0.010

Nagelkerke R^2^ = 0.252. Abbreviations: JIA, juvenile idiopathic arthritis; hsCRP, high-sensitivity C-reactive protein; JADAS27: Juvenile Arthritis Disease Activity Score, quantitative; bDMARD, biological disease-modifying antirheumatic drug; PAQ-C/PAQ-A, Physical Activity Questionnaire (children and adolescents). Variables included in the equation: age, sex, JADAS27, duration of bDMARD, hsCRP, physical activity PAQ-C/PAQA.

**Table 4:** Linear regression model of factors associated with FMI in patients with JIA.

Variable	Univariate B (95% CI)	Multivariate B (95% CI)	*p* Value
Age, years	0.545 (0.910, 1.952)	0.301 (0.167, 1.411)	0.014
Sex, female	0.278 (0.958, 9.625)		
Time since diagnosis, months	0.382 (0.393, 1.466)		
JADAS27	0.324 (0.249,1.371)	0.448 (0.163, 1.080)	0.009
Time with DMARDs, months	0.251 (0.050, 1.118)		
hsCRP, mg/L	0.284 (0.168, 1.625)		
Physical activity, PAQ-C/PAQ-A, mean (SD)	−0.438 (−0.879, −3.045)	−0.224 (−5.758, −0.287)	0.031

Nagelkerke R^2^ = 0.430. Abbreviations: JIA, juvenile idiopathic arthritis; hsCRP, high-sensitivity C-reactive protein; JADAS27, Juvenile Arthritis Disease Activity Score, quantitative; DMARD, disease-modifying antirheumatic drug; PAQ-C/PAQ-A, Physical Activity Questionnaire (children and adolescents). Variables included in the equation: age, sex, JADAS27, duration of bDMARD, hsCRP, physical activity PAQ-C/PAQ-A.

## Supplementary Materials

The following are available online at https://www.mdpi.com/article/10.3390/jcm10173949/s1, Supplementary Table S1: Anthropometric characteristics and body composition of patients with JIA and controls on the reference date, Table S2: Anthropometric characteristics and body composition of patients with JIA and high and low disease activity excluding patients with systemic JIA, Table S3: Factors associated with overweight/obesity in patients with JIA, Table S4: Logistic regression model of factors associated with overweight/obesity in patients with JIA excluding patients with systemic JIA, Table S5: Correlation between fat mass index, fat mass, and lean mass and characteristics of patients with JIA, Table S6: Linear regression model of factors associated with FMI in patients with JIA analysis excluding patients with systemic JIA.
