# Supplementary material for: Adiposity Is Related to Inflammatory Disease Activity in Juvenile Idiopathic Arthritis
Source: J Clin Med. 2021 Aug 31;10(17):3949. doi: 10.3390/jcm10173949 (PMC8432058; doi:10.3390/jcm10173949)
Supplement: Supplementary file 1 [file jcm-10-03949-s001.zip › jcm-1367014-supplementary.pdf]

Supplementary Materials

Table S1. Anthropometric characteristics and body composition of patients with JIA and controls on the reference date.

Anthropometric characteristic	JIA (n = 80)	Controls (n = 80)	*p* Value
Anthropometric measurement			
Weight, mean in kg (SD)	38.4 (16.0)	38.7 (14.3)	0.888
Height, mean in cm (SD)	141.8 (19.8)	143.0 (19.7)	0.554
BMI			
Mean in kg/m2 (DS)	18.2 (4.2)	18.1 (2.9)	0.936
Percentile, mean (SD)	42.0 (29.9)	46.4 (26.8)	0.329
SDS, mean (SD)	- 0.10 (1.3)	-0.10 (0.8)	0.994
BMI classification (WHO)			0.515
Extreme underweight, n (%)	0 (0.0)	0 (0.0)	
Underweight, n (%)	0 (0.0)	1 (1.3)	
Normal weight, n (%)	67 (83.8)	70 (87.5)	
Overweight, n (%)	10 (12.5)	8 (10.0)	
Obesity, n (%)	3 (3.8)	1 (1.3)	
Body composition measured by DXA			
Total fat mass (kg), mean (SD)	11.2 (8.9)	12.2 (7.5)	0.449
FMI (kg/m2), mean (SD)	5.5 (3.5)	5.6 (2.6)	0.760
Total lean mass (kg), mean (SD)	25.4 (9.2)	25.0 (8.8)	0.793
FFMI (kg/m2), mean (SD)	12.2 (2.1)	11.9 (1.5)	0.294
Fat mass, arms (kg), mean (SD)	1.0 (0.9)	1.1 (0.6)	0.287
Fat mass, legs (kg), mean (SD)	4.5(3.4)	3.7 (2.3)	0.265
Fat mass, trunk (kg), mean (SD)	5.1 (4.7)	5.2(3.0)	0.464
Lean mass, arms (kg), mean (SD)	2.2 (1.1)	2.1 (0.7)	0.157
Lean mass, legs (kg), mean (SD)	8.1 (3.3)	7.3 (3.3)	0.130
Lean mass, trunk (kg), mean (SD)	11.5 (4.4)	10.5 (4.0)	0.117

Abbreviations: JIA, juvenile idiopathic arthritis; SD, standard deviation; kg, kilogram; BMI, body mass index; SDS, score standard deviation; DXA, dual-energy x-ray absorptiometry; FMI, fat mass index; FFMI, fat-free mass index.

Table S2. Anthropometric characteristics and body composition of patients with JIA and high and low disease activity excluding patients with systemic JIA.

Anthropometric characteristic	JIA High activity (n = 13)	JIA Not high activity* (n = 58)	Controls (n = 80)	*p* Value
Anthropometric characteristics				
Weight, mean kg (SD)	43.9 (14.3)	37.4 (13.3)	38.7 (14.3)	0.382
Height, mean cm (SD)	148.3 (15.4)	144.3 (18.9)	143.0 (19.7)	0.744
BMI	20.7 (6.4)	17.6 (3.4)	18.1 (2.9)	0.016
Classification (WHO)				0.470
Normal weight, n (%)	10 (76.9)	49 (84.5)	71 (88.8)	
Overweight/Obesity, n (%)	3 (23.1)	9 (15.5)	9 (11.3)	
Body composition by DXA				
SAMMI, mean (SD)	5.3 (1.2)	5.0 (0.7)	4.3 (0.7)	<0.001
Total fat mass (kg), mean (SD)	17.8 (14.3)	10.1 (7.0)	12.2 (7.5)	0.015
FMI (kg/m2), mean (SD)	8.9 (5.0)	4.7 (2.8)	5.6 (2.6)	0.001
Total lean mass (kg), mean (SD)	27.8 (12.2)	24.9 (8.1)	25.0 (8.8)	0.589

FFMI (kg/m2), mean (SD)	13.0 (2.4)	11.8 (2.0)	11.9 (1.5)	0.210
Fat mass arms (kg), mean (SD)	1.5 (1.5)	0.8 (0.8)	1.1 (0.6)	0.020
Fat mass legs (kg), mean (SD)	6.4 (5.7)	3.9 (2.6)	3.7 (2.3)	0.008
Fat mass trunk (kg), mean (SD)	8.3 (8.6)	4.2 (3.5)	5.2(3.0)	0.004
Lean mass arms (kg), mean (SD)	2.4 (1.4)	2.3 (1.2)	2.1 (0.7)	0.139
Lean mass legs (kg), mean (SD)	8.6 (4.4)	8.0 (2.7)	7.3 (3.3)	0.249
Lean mass trunk (kg), mean (SD)	12.9 (5.8)	11.4 (3.6)	10.5 (4.0)	0.113
Physical activity				
PAQ-C/ PAQ-A (score), mean (SD)	2.5 (0.6)	2.6 (0.5)		0.784

Abbreviations: JIA, juvenile idiopathic arthritis; SD, standard deviation; RF, rheumatoid factor; ANA, antinuclear antibodies; hsCRP, high-sensitivity C-reactive protein; ESR, erythrocyte sedimentation rate; JADAS27, Juvenile Arthritis Disease Activity Score; CHAQ, Childhood Health Assessment Questionnaire; csDMARD, conventional synthetic disease-modifying antirheumatic drug; bDMARD, biologic DMARD; kg, kilogram; DXA, dual-energy x-ray absorptiometry; FMI, fat mass index; FFMI, fat-free mass index; PAQ-C/ PAQ-A, Physical Activity Questionnaire (children and adolescents). * Not high activity includes patients with moderate and mild activity and inactive patients according to JADAS27.

Table S3. Factors associated with overweight/obesity in patients with JIA.

**Variable**	**JIA overweight/obesity (n = 13)**	**JIA normal weight (n = 67)**	***p* Value**
Epidemiological			
Sex, female n (%)	7 (53.8)	49 (73.1)	0.165
Age, mean (SD)	9.7 (3.1)	10.9 (3.2)	0.241
Clinical laboratory			
Time since diagnosis of JIA, years, mean (SD)	6.8 (4.0)	6.4 (3.5)	0.721
Type of JIA			0.135
Systemic, n (%)	1 (7.7)	8 (11.9)	
Oligoarticular persistent, n (%)	4 (30.8)	34 (50.7)	
Oligoarticular extensive n (%)	3 (23.1)	10 (14.9)	
Polyarticular RF-positive, n (%)	1 (7.7)	0 (0.0)	
Polyarticular RF-negative, n (%)	4 (30.8)	15 (22.4)	
Uveitis, n (%)	4 (30.8)	16 (23.9)	0.600
Disease control			
CRP (mg/l), mean (SD)	3.7 (2.2)	3.9 (6.3)	0.688
hsCRP (mg/l), mean (SD)	4.2 (2.9)	1.9 (3.3)	0.048
ESR (mm/h), mean (SD)	12.2 (6.5)	8.0 (6.5)	0.066
JADAS27, mean (SD)	2.4 (3.8)	1.8 (4.0)	0.676
High activity, n (%)	4 (30.8)	11 (16.4)	0.225
Not high activity*, n (%)	9 (69.2)	56 (83.6)	0.225
Duration of activity, mean (SD)	116.7 (76.4)	78.2 (37.1)	0.079
CHAQ, mean (SD)	0.03 (0.1)	0.00 (0.0)	0.677
Treatment			
Current treatment			
csDMARDs, n (%)	6 (46.2)	36 (53.7)	0.617
bDMARD, n (%)	7 (53.8)	17 (25.4)	0.040
Type of DMARD			0.038
Anti-IL-1, n (%)	0 (0.0)	4 (23.5)	
Anti-IL-6, n (%)	2 (28.6)	0 (0.0)	
Anti-TNF-α, n (%)	5 (71.4)	13 (76.5)	

Cumulative dose of glucocorticoids (mg/kg), median (IQR)	105.2 (4.4–31.2)	4.4 (2.4–14.4)	0.102
Duration of treatment			
Duration of csDMARDs (months), mean (SD)	52.0 (33.9)	50.8 (36.2)	0.912
Duration of bDMARDs (months), mean (SD)	37.1 (20.2)	16.2 (14.4)	0.014
Total duration of DMARDs (months), mean (SD)	54.1(35.3)	56.1 (36.7)	0.861
Body composition by DXA			
Total fat mass (kg), mean (SD)	11.3 (0.9)	10.5 (8.4)	0.779
FMI (kg/m2), mean (SD)	5.5 (3.6)	5.1 (3.7)	0.733
Total lean mass (kg), mean (SD)	23.3 (7.4)	25.8 (9.5)	0.442
FFMI (kg/m2), mean (SD)	12.2 (2.3)	12.3 (1.0)	0.836
Fat mass arms (kg), mean (SD)	1.0 (1.2)	0.9 (0.8)	0.848
Fat mass legs (kg), mean (SD)	4.3 (1.4)	4.2 (3.3)	0.913
Fat mass trunk (kg), mean (SD)	5.2 (4.4)	4.6 (3.6)	0.711
Fat mass gynoid (kg), mean (SD)	2.1 (1.9)	2.1 (1.5)	0.943
Fat mass android (kg) mean (SD)	0.8 (1.0)	0.7 (0.6)	0.488
Lean mass arms (kg), mean (SD)	2.0 (0.7)	2.4 (1.3)	0.246
Lean mass legs (kg), mean (SD)	7.3 (2.4)	8.3 (3.4)	0.352
Lean mass trunk (kg), mean (SD)	11.7 (4.1)	11.9 (4.5)	0.898
Lean mass gynoid (kg), mean (SD)	2.7 (0.9)	3.1 (1.5)	0.178
Lean mass android (kg), mean (SD)	1.4 (0.3)	1.4 (0.5)	0.677
Physical activity			
PAQ-C/PAQ-A, mean (SD)	2.6 (0.6)	2.9 (0.5)	0.080

Abbreviations: JIA, juvenile idiopathic arthritis; SD, standard deviation; RF, rheumatoid factor; ANA, antinuclear antibodies; hsCRP, high-sensitivity C-reactive protein; ESR, erythrocyte sedimentation rate; JADAS27, Juvenile Arthritis Disease Activity Score; CHAQ, Childhood Health Assessment Questionnaire; csDMARD, conventional synthetic disease-modifying antirheumatic drug; bDMARD, biologic DMARD; kg, kilogram; DXA, dual-energy x-ray absorptiometry; FMI, fat mass index; FFMI, fat-free mass index; PAQ-C/ PAQ-A, Physical Activity Questionnaire (children and adolescents). * Not high activity includes patients with moderate and mild activity and inactive patients according to JADAS27.

Table S4. Logistic regression model of factors associated with overweight/obesity in patients with JIA excluding patients with systemic JIA

Variable	Univariate OR (95% CI)	Multivariate OR (95% CI)	*p* Value
Age, years	0.868 (0.719, 1.048)		
Sex, female	0.357 (0.096, 1.326)		
Disease duration, months	1.005 (0.998, 1.013)		
JADAS27	1.009 (0.871, 1.168)		
Duration of bDMARD, months	1.026 (1.006, 1.046)	1.030 (1.008, 1.053)	0.027
hsCRP, mg/l	1.124 (0.993, 1.272)		
Physical activity, PAQ-C/PAQ-A, mean (SD)	0.410 (0.150, 1.120)	0.200 (0.058, 0.680)	0.036

Abbreviations: JIA, juvenile idiopathic arthritis; hsCRP, high-sensitivity C-reactive protein; JADAS27: Juvenile Arthritis Disease Activity Score, quantitative; bDMARD, biological disease-modifying antirheumatic drug; PAQ-C/ PAQ-A, Physical Activity Questionnaire (children and adolescents). Variables included in the equation: age, sex, JADAS27, duration of bDMARD, hsCRP, physical activity PAQ-C/PAQA.

Table S5. Correlation between fat mass index, fat mass, and lean mass and characteristics of patients with JIA.

Variable	Fat mass Spearman *p*	FMI Spearman *p*	Lean mass Spearman *p*
Age, years	0.350**	0.545**	0.774**

BMI percentile	0.028*	0.066*	–0.130*
Time since diagnosis, months	0.263**	0.382**	0.463***
JADAS27	0.395**	0.394**	0.0109*
CHAQ	0.185*	0.198*	0.135*
CRP, mg/l	0.212*	0.099*	–0.077*
hsCRP, mg/l	0.194*	0.260**	–0.180*
ESR, mm/h	0.201*	0.121*	–0.121*
Time with csDMARDs, months	0.137*	0.269**	0.456***
Time with bDMARDs, months	0.012*	0.040*	0.176*
Time with DMARDs, months	0.137*	0.251**	0.456***
Time with glucocorticoids, months	0.118*	0.111*	0.132*
Physical activity, PAQ-C/PAQ-A, mean (SD)	–0.438***	–0.438***	0.317**

*$p > 0.05$; **$p < 0.05$; ***$p < 0.001$. Abbreviations; JIA, juvenile idiopathic arthritis; SD, standard deviation; BMI, body mass index; hsCRP, high-sensitivity C-reactive protein; JADAS27: Juvenile Arthritis Disease Activity Score, quantitative; CHAQ, Childhood Health Assessment Questionnaire; ESR, erythrocyte sedimentation rate; csDMARD, conventional synthetic disease-modifying antirheumatic drug; bDMARD, biologic DMARD; PAQ-C/ PAQ-A, Physical Activity Questionnaire (children and adolescents).

Table S6. Linear regression model of factors associated with FMI in patients with JIA analysis excluding patients with systemic JIA.

Variable	Univariate B (95% CI)	Multivariate B (95%CI)	*p* Value
Age, years	0.399 (0.081, 0.716)	0.290 (0.150, 1.300)	0.019
Sex, female	0.340 (0.298, 5.666)		
Time since diagnosis, months	0.272 (-0.009, 0.553)		
JADAS27	0.343 (0.196,0.950)	0.339 (0.107, 0.570)	0.015
Time with DMARDs, months	0.194 (0.045, 1.008)		
hsCRP, mg/l	0.106 (0.100, 1.612)		
Physical activity, PAQ-C/PAQ-A, mean (SD)	–0.399 (–0.716, –0.410)	–0.246 (–3.869, –0.151)	0.036

Nagelkerke $R^2 = 0.400$. Abbreviations: JIA, juvenile idiopathic arthritis; hsCRP, high-sensitivity C-reactive protein; JADAS27, Juvenile Arthritis Disease Activity Score, quantitative; DMARD, disease-modifying antirheumatic drug; PAQ-C/ PAQ-A, Physical Activity Questionnaire (children and adolescents). Variables included in the equation: age, sex, JADAS27, duration of bDMARD, hsCRP, physical activity PAQ-C/PAQ-A.
